# Ca^2+^ Dependence of Volume-Regulated VRAC/LRRC8 and TMEM16A Cl^–^ Channels

**DOI:** 10.3389/fcell.2020.596879

**Published:** 2020-12-01

**Authors:** Raquel Centeio, Jiraporn Ousingsawat, Rainer Schreiber, Karl Kunzelmann

**Affiliations:** Physiological Institute, University of Regensburg, Regensburg, Germany

**Keywords:** VRAC, CaCC, TMEM16 proteins, anoctamin, ANO1

## Abstract

All vertebrate cells activate Cl^–^ currents (I_Cl__,swell_) when swollen by hypotonic bath solution. The volume-regulated anion channel VRAC has now been identified as LRRC8/SWELL1. However, apart from VRAC, the Ca^2+^-activated Cl^–^ channel (CaCC) TMEM16A and the phospholipid scramblase and ion channel TMEM16F were suggested to contribute to cell swelling-activated whole-cell currents. Cell swelling was shown to induce Ca^2+^ release from the endoplasmic reticulum and to cause subsequent Ca^2+^ influx. It is suggested that TMEM16A/F support intracellular Ca^2+^ signaling and thus Ca^2+^-dependent activation of VRAC. In the present study, we tried to clarify the contribution of TMEM16A to I_Cl__,swell_. In HEK293 cells coexpressing LRRC8A and LRRC8C, we found that activation of I_Cl__,swell_ by hypotonic bath solution (Hypo; 200 mosm/l) was Ca^2+^ dependent. TMEM16A augmented the activation of LRRC8A/C by enhancing swelling-induced local intracellular Ca^2+^ concentrations. In HT_29_ cells, knockdown of endogenous TMEM16A attenuated I_Cl__,swell_ and changed time-independent swelling-activated currents to VRAC-typical time-dependent currents. Activation of I_Cl__,swell_ by Hypo was attenuated by blocking receptors for inositol trisphosphate and ryanodine (IP_3_R; RyR), as well as by inhibiting Ca^2+^ influx. The data suggest that TMEM16A contributes directly to I_Cl__,swell_ as it is activated through swelling-induced Ca^2+^ increase. As activation of VRAC is shown to be Ca^2+^-dependent, TMEM16A augments VRAC currents by facilitating Hypo-induced Ca^2+^ increase in submembraneous signaling compartments by means of ER tethering.

## Introduction

Cell swelling activates Cl^–^ currents (I_Cl__,swell_) that are known as volume*-*regulated anion channels (VRAC) (Jentsch, 2016; Pedersen et al., 2016; Strange et al., 2019). VRAC is now identified as LRRC8/SWELL1 (Qiu et al., 2014; Voss et al., 2014). Its hexameric structure has been reported in four independent cryo-EM studies (Deneka et al., 2018; Kasuya et al., 2018; Kefauver et al., 2018; Kern et al., 2019). The essential subunit LRRC8A along with a variable combination of additional four paralogous proteins LRRC8B-E forms heteromers with different kinetic properties. Numerous studies show a contribution of these channels to cellular resistance toward cisplatin and other anticancer drugs (Ise et al., 2005; Mahmud et al., 2008; Planells-Cases et al., 2015) and possibly growth of cancer (Lemonnier et al., 2004; Kunzelmann et al., 2019; Liu and Stauber, 2019;Lu et al., 2019). Others reported a role of VRAC in apoptotic cell death (Gulbins et al., 2000; Okada et al., 2006; Wanitchakool et al., 2016). Despite structural information, the mechanism for activation of VRAC/LRRC8 channels remains incompletely understood. While lowering intracellular ionic strength is an essential prerequisite (Cannon et al., 1998; Sabirov et al., 2000; Syeda et al., 2016), intracellular Ca^2+^ was shown to be important for activation of I_Cl__,swell_ and for cellular volume regulation (Mccarty and O’neil, 1992; Lemonnier et al., 2002; Akita et al., 2011; Benedetto et al., 2016; Liu et al., 2019).

Cell swelling activates not only VRAC/LRRC8 but also Ca^2+^-dependent TMEM16A (Almaca et al., 2009; Benedetto et al., 2016; Liu et al., 2019) and TMEM16F channels/scramblases (Juul et al., 2014; Sirianant et al., 2016a). Our earlier results show that both TMEM16A and LRRC8 contribute to I_Cl__,swell_ (Benedetto et al., 2016) in a Ca^2+^-dependent manner. This has been shown also for serum-induced current activation (Zhang et al., 2020). In fact, we demonstrated that LRRC8 is not essential for I_Cl__,swell_ or cellular volume regulation (Milenkovic et al., 2015; Sirianant et al., 2016b). Particularly in differentiated naïve cells in tissues, and in epithelial cells with large transport capacity, the physiological relevance of LRRC8 may be limited (Almaca et al., 2009; Milenkovic et al., 2015; Sirianant et al., 2016b).

Previous studies did not answer the question whether LRRC8 and TMEM16A/F are activated in parallel to give rise to I_Cl__,swell_, or whether TMEM16 proteins, particularly TMEM16A, support activation of LRRC8 by facilitating Ca^2+^ signals near the plasma membrane (Benedetto et al., 2016; Cabrita et al., 2017). For comparison, such a Ca^2+^-modulating effect of TMEM16A is fundamental for activation of CFTR as demonstrated *in vitro* and *in vivo* in mouse and human (Von Kleist et al., 1975; Benedetto et al., 2017, 2019b; Park et al., 2020), which express both proteins endogenously. The data provide evidence that TMEM16A supports Ca^2+^ release from endoplasmic reticulum (ER) and cause Ca^2+^ influx that activates TMEM16A and supports activation of LRRC8.

## Materials and Methods

### Cells Culture

All cells were grown at 37°C in a humidified atmosphere with 5% (v/v) CO_2_. Human embryonic kidney HEK293T cells (Simmons, 1990) stably expressing iodide-sensitive enhanced yellow fluorescent protein (eYFP-I152L; HEK-YFP; Amgen, Thousand Oaks, California, United States) were grown in DMEM low-glucose medium supplemented with 10% (v/v) fetal bovine serum (FBS), 1% (v/v) L-glutamine 200 mM, and 10 mM HEPES (all from Capricorn Scientific, Ebsdorfergrund, Germany), in the presence of selection antibiotic puromycin 0.5 μg/mL (Sigma-Aldrich, Missouri, United States). Cells stably coexpressing TMEM16A (HEK-YFP-TMEM16A) were cultured under additional hygromycin B (150 μg/mL; Capricorn Scientific, Ebsdorfergrund, Germany). HT29 human colonic carcinoma epithelial cells stably expressing eYFP-I152L (HT29-YFP, kindly provided by Prof. Luis Galietta, TIGEM, Pozzuoli, and Prof. Nicoletta Pedemonte, Istituto Giannina Gaslini, Genua, Italy.) were cultured in McCoy’s 5A medium supplemented with 10% (v/v) FBS and 1 mg/mL G418 selection antibiotic (all from Capricorn Scientific, Ebsdorfergrund, Germany).

### RT-PCR, siRNA, and cDNAs

Human LRRC8A (NM_00127244.1) and LRRC8C (NM_032270.5) were sub-cloned into the bicistronic vector pIRES2 (Clontech Laboratories/Takara Bio United States, Mountain View, California, United States) using standard methods. The construct was verified by sequencing (Microsynth Seqlab, Göttingen, Germany). Transfection of cDNAs into HEK293T cells was performed using standard protocols for Lipofectamine 3000 (Invitrogen, Carlsbad, California, United States). CD8 cDNA was co-transfected to allow for detection of overexpressing cells by binding of anti-CD8 labeled beads (Dynabeads^®^ M-450 Epoxy; Invitrogen, Carlsbad, California, United States). Knockdown of TMEM16A and LRRC8A in HT29 cells was performed through transfection by electroporation of the siRNAs siTMEM16A (5-GGUUCCCAG CCUACCUCACUAACUU-3, Invitrogen, Carlsbad, California, United States) and/or siLRRC8A (5-CCAAGC UCAUCGUCCUCAA-3, Ambion, Austin, Texas, United States), using a Neon Transfection System (Invitrogen, Carlsbad, California, United States) with a program of three pulses, 1,650 V, and 10 ms. Scrambled siRNA (Silencer^®^ Select Negative Control siRNA #1, Ambion, Austin, Texas, United States) was transfected as negative control. All experiments were performed 48–72 h after transfection.

### Western Blotting

Protein was isolated from cells using a lysing buffer containing 25 mM Tris–HCl pH 7.4, 150 mM NaCl, 1 mM EDTA, 5% glycerol, 0.43% Nonidet P-40, 100 mM dithiothreitol (both from PanReac AppliChem, Barcelona, Spain), and 1× protease inhibitor mixture (Roche, Basel, Switzerland). Proteins were then separated by an 8.5% SDS-PAGE and transferred to a PVDF membrane (GE Healthcare, Munich, Germany). Membranes were incubated with primary rabbit monoclonal anti-TMEM16A/DOG1 antibody (#NBP1-49799; Novus Biologicals, Littleton, Colorado, United States; 1:500 in 1% (w/v) NFM/TBS-T) or rabbit polyclonal anti-LRRC8A antibody (#HPA016811; Sigma-Aldrich, St. Louis, Missouri, United States; 1:1,000 in 1% (w/v) BSA/TBS-T) for 2.5 h at room temperature. A rabbit polyclonal anti-β-actin antibody (A2066; Sigma-Aldrich, St. Louis, Missouri, United States; 1:10,000 in 5% (w/v) NFM/TBS-T) was used for loading control. Membranes were then incubated with horseradish peroxidase (HRP)-conjugated goat polyclonal anti-rabbit secondary antibody (#31460; Invitrogen, Carlsbad, California, United States) at RT for 2 h, and immunoreactive signals were visualized using a SuperSignal Chemiluminescent Substrate detection kit (#34577; Thermo Fisher Scientific, Waltham, Massachusetts, United States).

### Patch Clamping

Cells were patch clamped on glass cover slips. If not indicated otherwise, patch pipettes were filled with a cytosolic-like solution containing (mM) KCl 30, K-gluconate 95, NaH2PO4 1.2, Na2HPO4 4.8, EGTA 1, Ca-gluconate 0.758, MgCl2 1.03, D-glucose 5, ATP 3; 290 mosm/l; pH 7.2. The free Ca^2+^ concentration (Ca^2+^ activity) was 0.1 μM. To adjust pipette Ca^2+^ concentrations to 0.01 μM, the pipette-filling solution contained (mM) KCl 30, K-gluconate 95, NaH2PO4 1.2, Na2HPO4 4.8, EGTA 1, Ca-gluconate 0.209, MgCl2 1.03, D-glucose 5, and ATP 3; 290 mosm/l; pH 7.2. Free Ca^2+^ concentrations were calculated according to a program developed at the Max Planck institute for biophysics (Frankfurt, Germany). Ca^2+^ activities (free Ca^2+^ concentrations) were originally validated by potentiometric determination using Ca^2+^-sensitive electrodes. In some experiments, patch pipettes were filled with CsCl buffer containing (mM) CsCl 125, NaH2PO4 1.2, Na2HPO4 4.8, EGTA 1, Ca-gluconate 0.209, MgCl2 1.03, D-glucose 5, and ATP 3; pH 7.2. In these experiments, a CsCl buffer was used as bath solution, containing 145 CsCl, 0.4 KH2PO4, 1.6 K2HPO, 4.6 D-glucose, 1 MgCl2, and 1.3 Ca^2+^ gluconate, 290 mosm/l; pH 7.4.

Coverslips were mounted in a perfused bath chamber on the stage of an inverted microscope (IM35, Zeiss) and kept at 37°C. The bath was perfused continuously with Ringer solution at a rate of 8 ml/min. For activation of volume-dependent Cl^–^ currents, isotonic Ringer bath solution (300 mosm/l; mM: 145 NaCl, 0.4 KH2PO4, 1.6 K2HPO, 4.6 D-glucose, 1 MgCl2, 1.3 Ca^2+^ gluconate, pH 7.4) was changed to hypotonic bath solution (Hypo; 200 mosm/l) by removing 50 mM NaCl from Ringer solution. Patch-clamp experiments were performed in the fast whole-cell configuration. Patch pipettes had an input resistance of 4–6 MΩ when filled with the cytosolic-like (physiological) solution. Currents were corrected for serial resistance. The access conductance was measured continuously and was 60–140 nS. Currents (voltage clamp) and voltages (current clamp) were recorded using a patch-clamp amplifier (EPC 7, List Medical Electronics, Darmstadt, Germany), the LIH1600 interface, and PULSE software (HEKA, Lambrecht, Germany) as well as Chart software (AD Instruments, Spechbach, Germany). Data were stored continuously on a computer hard disc and analyzed using PULSE software. In regular intervals, membrane voltage (Vc) was clamped in steps of 20 mV from −100 to +100 mV from a holding voltage of −100 mV. Current density was calculated by dividing whole-cell currents by cell capacitance.

### YFP Quenching

Quenching of the intracellular fluorescence generated by stable transfection of the iodide (I^–^)-sensitive enhanced yellow fluorescent protein (eYFP-I152L) was used to measure anion conductance. YFP fluorescence was excited at 490 nm using a high-speed polychromatic illumination system for microscopic fluorescence measurements (Visitron Systems, Puchheim, Germany), and the emitted light at 535 ± 25 nm was detected with a CoolSnap HQ CCD camera (Roper Scientific, Planegg, Germany/Visitron Systems, Puchheim, Germany). Cells were grown on coverslips and mounted in a thermostatically controlled imaging chamber adapted to an inverted microscope (Axiovert S100, Zeiss, Oberkochen, Germany), maintained at 37°C, and perfused at a rate of 5 mL/min. After a brief period under standard Ringer solution (in mM: NaCl 145, KH_2_PO_4_ 0.4, K_2_HPO_4_ ⋅ 3 H_2_0 1.6, glucose 5, MgCl_2_ ⋅ 6 H_2_0 1, Ca-gluconate ⋅ 1 H_2_0 1.3), cells were stimulated with hypotonic (200 mosm/l) solution with the following composition (in mM): NaCl 45, KH_2_PO_4_ 0.4, K_2_HPO_4_ ⋅ 3 H_2_0 1.6, glucose 5, MgCl_2_ ⋅ 6 H_2_0 1, Ca-gluconate ⋅ 1 H_2_0 1.3, Na-gluconate 50) or isotonic solution as control (in mM: NaCl 45, KH_2_PO_4_ 0.4, K_2_HPO_4_ ⋅ 3 H_2_0 1.6, glucose 5, MgCl_2_ ⋅ 6 H_2_0 1, Ca-gluconate ⋅ 1 H_2_0 1.3, Na-gluconate 50, mannitol 100). Quenching of YFP-fluorescence by I^–^ influx was induced by replacing 50 mM extracellular gluconate by I^–^. Control of experiment, imaging acquisition, and data analysis were done with the software package MetaFluor (Universal Imaging, Bedford Hills, New York, United States). Autofluorescence was negligible. For quantitative analysis, cells with low or excessively high fluorescence were discarded. Changes in fluorescence induced by I^–^ uptake are expressed as initial rates of maximal fluorescence decrease (au/s).

Alternatively, YFP-quenching measurements were performed on a fluorescence microplate reader (NOVOstar, BMG Labtech, Ortenberg, Germany) kept at 37°C, using an excitation wavelength of 485 nm and emission detection at 520 nm. Cells were plated in transparent 96-well plates (Sarstedt, Nümbrecht, Germany), cultured 48–72 h to 80–90% confluence, and incubated with or without test compounds in standard Ringer solution (300 mosm/l). After a short basal fluorescence reading, cells were stimulated with a hypotonic (200 mosm/l) solution (in mM: NaCl 21.67, NaI 26.67, KH_2_PO_4_ 0.4, K_2_HPO_4_ ⋅ 3 H_2_0 1.6, glucose 5, MgCl_2_ ⋅ 6 H_2_0 1, Ca-gluconate ⋅ 1 H_2_0 1.3, Mannitol 58) added by automated injection through a syringe pump, or isotonic solution (in mM: NaCl 21.67, NaI 26.67, KH_2_PO_4_ 0.4, K_2_HPO_4_ ⋅ 3 H_2_0 1.6, glucose 5, MgCl_2_ ⋅ 6 H_2_0 1, Ca-gluconate ⋅ 1 H_2_0 1.3, mannitol 193.3) as control. For ATP stimulation, cells were incubated with or without test compounds in a gluconate-substituted Ringer solution (in mM: NaCl 100, Na-gluconate 40, KCl 5, MgCl_2_ ⋅ 6 H_2_0 1, CaCl_2_ ⋅ 2 H_2_0 2, glucose 10, HEPES 10) and ATP was added in a symmetrical I^–^-substituted Ringer solution (in mM: NaCl 100, NaI 40, KCl 5, MgCl_2_ ⋅ 6 H_2_0 1, CaCl_2_ ⋅ 2 H_2_0 2, D-glucose 10, HEPES 10). The final I^–^ concentration on each well was 20 mM for every experiment. Total intracellular YFP-fluorescence intensity in each well was measured continuously. Background fluorescence was subtracted, and data was normalized to the initial fluorescence. The initial rate of maximal fluorescence decay caused by I^–^ influx was then calculated as a measure of anion conductance.

### Ca^2+^ Measurements

Cells were seeded on glass coverslips and loaded with 2 μM Fura-2, AM Ester (Biotium, Hayward, California, United States), and 0.02% Pluronic F-127 (Invitrogen, Carlsbad, California, United States) in standard Ringer solution (in mM: NaCl 145, KH_2_PO_4_ 0.4, K_2_HPO_4_ ⋅ 3 H_2_0 1.6, glucose 5, MgCl_2_ ⋅ 6 H_2_0 1, Ca-gluconate ⋅ 1 H_2_0 1.3) for 1 h at room temperature. Cells were then mounted in a thermostatically controlled imaging chamber adapted to an inverted microscope (Axiovert S100, Zeiss, Oberkochen, Germany), maintained at 37°C and perfused at a rate of 5 mL. Fura-2 was excited at 340/380 nm using a high-speed polychromatic illumination system for microscopic fluorescence measurements (Visitron Systems, Puchheim, Germany), and emission was recorded between 470 and 550 nm using a CoolSnap HQ CCD camera (Roper Scientific, Planegg, Germany/Visitron Systems, Puchheim, Germany). Cells were stimulated with ATP or hypotonic (200 mosm/l) solution (in mM: NaCl 95, KH_2_PO_4_ 0.4, K_2_HPO_4_ ⋅ 3 H_2_0 1.6, glucose 5, MgCl_2_ ⋅ 6 H_2_0 1, Ca-gluconate ⋅ 1 H_2_0 1.3). Intracellular calcium ([Ca^2+^ ]_i_) was calculated from the 340/380-nm fluorescence ratio after background subtraction using the formula *[Ca^2+^ ]_*i*_ = Kd × (R−R_*min*_)/(R_*max*_−R) × (S_*f2*_/S_*b2*_)*, where *R* is the observed fluorescence ratio. The values *R*_*max*_ and *R*_*min*_ (maximum and minimum ratios) and the constant *S_*f2*_/S_*b2*_* (fluorescence of free and Ca^2+^-bound Fura-2 at 380 nm) were calculated using 2 μM ionomycin (Calbiochem, San Diego, California, United States), 5 μM nigericin (Sigma-Aldrich, St. Louis, Missouri, United States), 10 μM monensin (Sigma-Aldrich, St. Louis, Missouri, United States), and 5 mM EGTA (Carl Roth, Karlsruhe, Germany) to equilibrate intracellular and extracellular Ca^2+^ in intact Fura-2-loaded cells. The dissociation constant (*Kd*) for the Fura-2⋅Ca^2+^ complex was taken as 224 nM. Control of experiment, imaging acquisition, and data analysis were done with the software package MetaFluor (Universal Imaging, Bedford Hills, New York, United States).

### Materials and Statistical Analysis

Suramin, U73122, 2-APB, dantrolene, and NS3728 were from Sigma-Aldrich, St. Louis, Missouri, United States. Niclosamide, Ani9, and DCPIB were from Tocris, Bristol, United Kingdom. Xestospongin C and niclosamide ethanolamine were from Cayman Chemical, Ann Arbor, Michigan, United States. Probenecid was from MP Biomedicals, Irvine, California, United States. Data are shown as individual traces or as summaries with mean values ± SEM and number of experiments given in each figure’s legend. For statistical analysis, paired or unpaired Student’s *t*-test was used as appropriate. A *p*-value of < 0.05 was accepted as a statistically significant difference (indicated by # for unpaired data and by ^∗^ for paired data).

## Results

### Activation of Endogenous and Overexpressed VRAC Is Ca^2+^ Dependent

**FIGURE 1:**
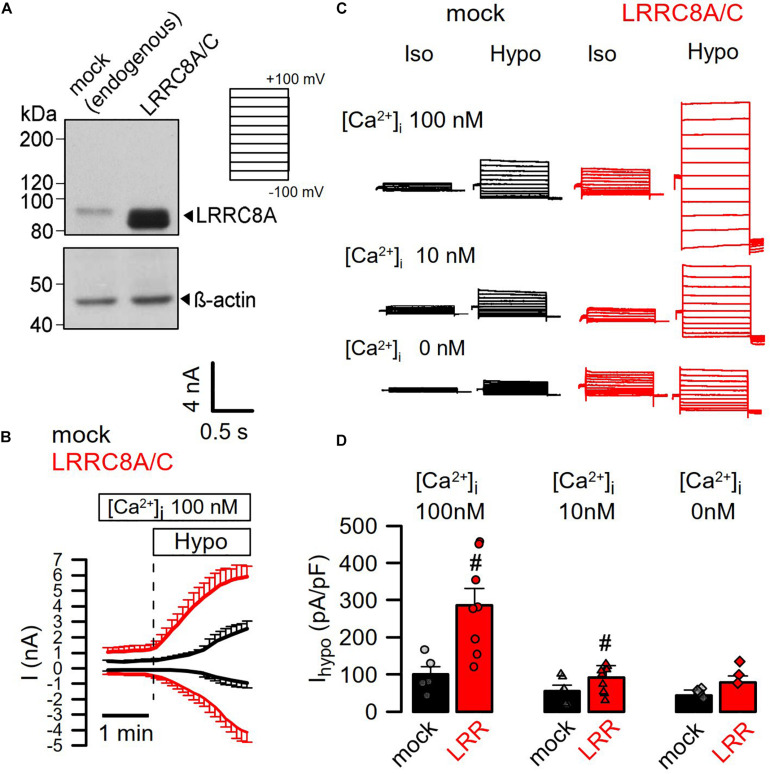
Activation of LRRC8A/C is Ca^2+^ dependent. **(A)** Coexpression of LRRC8A and LRRC8C by a bicistronic vector. Detection of LRRC8A by Western blotting. **(B)** Continuous recording of whole-cell currents activated by hypotonic cell swelling (Hypo, 200 mosm/l). Patch pipette [Ca^2+^ ] was 100 nM; extracellular Ringer [Ca^2+^ ] was 1.3 mM. **(C)** Whole-cell current overlays obtained with 100, 10, or 0 nM Ca^2+^ in the patch pipette filling solution. Clamp voltages ranged from −100 to +100 mV in steps of 20 mV. **(D)** Summary of Hypo-induced whole-cell current densities (Vc = +100 mV) obtained in mock-transfected and LRRC8A/C-expressing HEK293 cells (*p* < 0.001 and < 0.01, respectively). Mean ± SEM; *n* = 5–9 for each series). ^#^Significant increase when compared to mock (unpaired *t*-tests). Blots were done as replicates.

Earlier studies suggested a requirement of Ca^2+^ for activation of I_Cl__,swell_ and stimulation of endogenous LRRC8/Swell1 (Akita and Okada, 2011; Benedetto et al., 2016). Here we directly validated the Ca^2+^ dependence of LRRC8 currents by coexpressing both LRRC8A and LRRC8C in HEK293 cells, using a bicistronic expression plasmid. Figure 1A shows strong increase of LRRC8A expression when compared with endogenous LRRC8A expression (overexpression of LRRC8C not shown). Overexpression of LRRC8A/C largely augmented the whole-cell currents activated by hypotonic (200 mosm/l) bath solution (Hypo) in the presence of an intracellular (pipette, 290 mosm/l) Ca^2+^ concentration of 100 nM. Overexpressed LRRC8A/C was very rapidly activated by Hypo-induced cell swelling (Figure 1B). Gradual decrease of intracellular (i.e., patch pipette) Ca^2+^ concentrations to 10 and to 0 nM gradually inhibited hypo-activation of the endogenous I_Cl__,swell_, as well as the overexpressed LRRC8A/C currents (Figures 1C,D). The experiments indicate a requirement of Ca^2+^ for activation of VRAC/LRRC8.

### TMEM16A Supports Activation of I_Cl__,swell_ in HEK293 Cells Overexpressing LRRC8A/C

**FIGURE 2:**
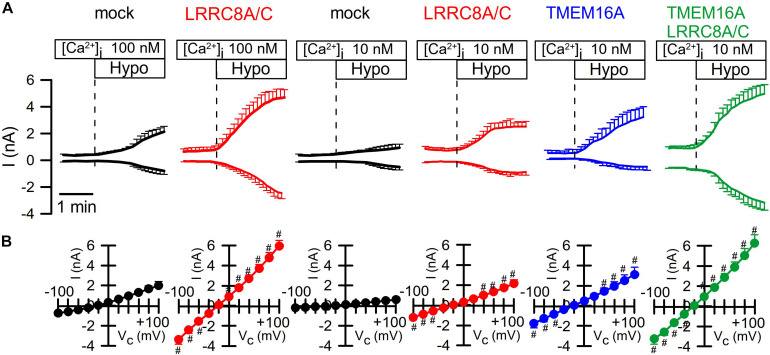
TMEM16A is activated by Hypo and augments I_Cl__,swell_. **(A)** Continues recording of whole cell currents activated in HEK293 cells by hypotonic cell swelling (Hypo, 200 mosm/l). **(B)** Current/voltage relationships of Hypo-activated whole-cell currents. TMEM16A is activated by Hypo and further augments I_Cl__,swell_ in cells coexpressing TMEM16A and LRRC8A/C. Mean ± SEM; *n* = 8–11 for each series). ^#^Significant increase when compared to mock (*p* < 0.05–0.0001; unpaired *t*-tests).

It has been reported that TMEM16A (and TMEM16F) take part in whole-cell current activated through hypotonic cell swelling (I_Cl__,swell_), although these proteins are not known to be directly activated by cell swelling (Almaca et al., 2009; Benedetto et al., 2016; Sirianant et al., 2016a; Liu et al., 2019). Here we examined the impact of TMEM16A on activation of VRAC at different intracellular Ca^2+^ concentrations. At an intracellular resting Ca^2+^ concentration of 100 nM, endogenous VRAC (mock) and overexpressed LRRC8A/C were readily activated by osmotic cell swelling (200 mosm/l; Hypo). However, activation was compromised at 10 nM intracellular Ca^2+^ ([Ca^2+^ ]_i_) (Figure 2A). Even at low (10 nM) [Ca^2+^ ]_i_, overexpression of TMEM16A increased hypotonic activation of endogenous VRAC and overexpressed LRRC8A/C (Figures 2A,B). This could suggest that Ca^2+^ influx is also relevant for activation of VRAC, as suggested earlier (Sirianant et al., 2016a).

**FIGURE 3:**
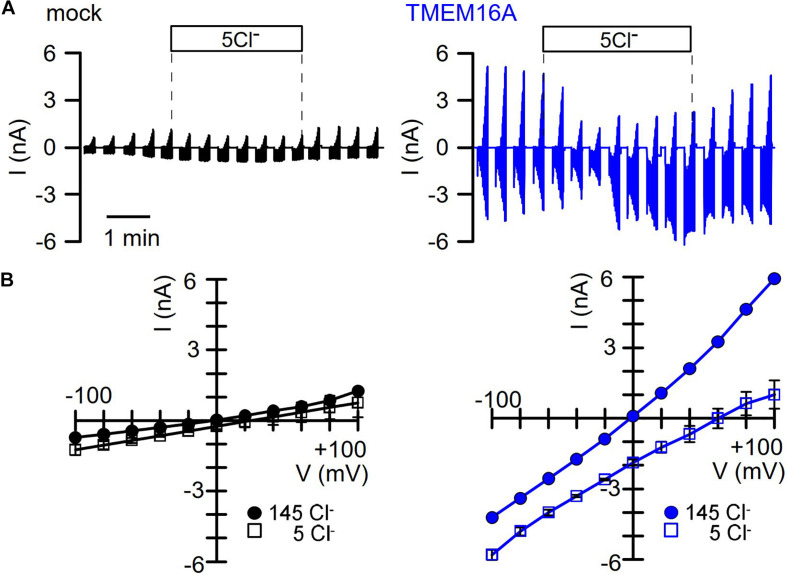
TMEM16A overexpressed in HEK293 cells is spontaneously active. **(A)** Continuous current recordings of a mock-transfected and a TMEM16A-expressing HEK293 cell. In these experiments, the pipette filling solution and the bath solution contained CsCl (c.f. Methods). Cl^–^ removal from the extracellular bath solution except of 5 mM (5Cl^–^) had very little effect on the mock-transfected cell but strongly depolarized the TMEM16A-expressing cell. **(B)** Corresponding I/V curves indicate small currents and little effect of 5Cl^–^ in mock-transfected cells, while currents were large and strongly inhibited with a shift of the reversal potential in TMEM16A-overexpressing cells.

We intended to perform similar experiments with overexpressed TMEM16A at 100 nM pipette Ca^2+^, to examine how the presence of TMEM16A would affect volume activation of VRAC. However, we found overexpressed TMEM16A to be active even at this basal intracellular Ca^2+^ concentration and without any additional Ca^2+^ increase by hormonal stimulation or by Ca^2+^ ionophores (Figure 3). In these experiments, we used CsCl buffer as patch pipette filling solution (100 nM Ca^2+^; 290 mosm/l) and an extracellular CsCl buffer (1.3 mM Ca^2+^; 300 mosm/l), to exclude any possible contribution of K^+^ currents. In contrast to mock-transfected cells, TMEM16A-overexpressing cells demonstrated a large basal Cl^–^ inward current that was inhibited by removal of extracellular Cl^–^, causing a right shift of the reversal potential (Figures 3A,B). Only overexpressed, but not endogenous TMEM16A, was found to be active at basal intracellular [Ca^2+^ ] levels, which has been examined in detail earlier (Sirianant et al., 2016a; Schreiber et al., 2018). Thus, it was difficult to assess the contribution of overexpressed TMEM16A to swelling activation of VRAC in HEK293 cells at [Ca^2+^ ]_i_ of 100 nM.

Overexpressed TMEM16A was not active under basal conditions with 10 nM intracellular Ca^2+^ (Figure 2). Overexpression of TMEM16A together with LRRC8A/C further augmented I_Cl__,swell_, but it remained unclear whether TMEM16A itself is activated during cell swelling (by Ca^2+^ store release or Ca^2+^ influx) or whether TMEM16A supports activation of LRRC8A/C. We therefore performed additional experiments in HT_29_ cells, which express both ion channels endogenously.

### TMEM16A Supports Activation of I_Cl__,swell_ in HT_2__9_ Cells Expressing Endogenous LRRC8 and TMEM16A

**FIGURE 4:**
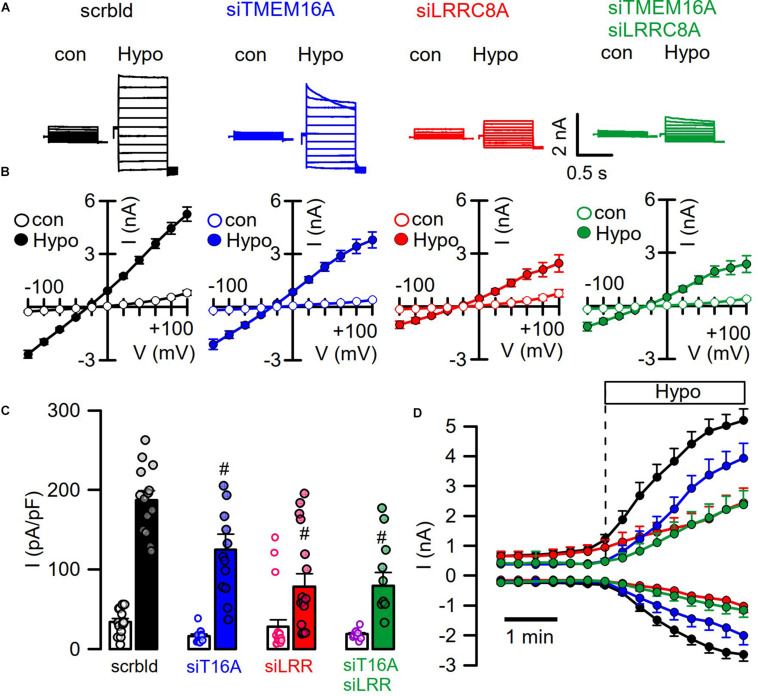
TMEM16A supports activation of LRRC8. **(A)** Hypo (200 mosm/l)-induced whole-cell current overlays obtained in HT_29_ cells treated with scrambled RNA (scrbld), siRNA for TMEM16A, LRRC8A, or both. The free Ca^2+^ concentration in the patch pipette filling solution was 100 nM. **(B)** Corresponding current–voltage relationships. **(C)** Hypo-induced whole-cell current densities (Vc = +100 mV). **(D)** Time-dependent activation of whole-cell currents by Hypo. Mean ± SEM; *n* = 9–14 for each series). ^#^Significant decrease when compared to mock (*p* < 0.01 for all; unpaired *t*-tests).

We reexamined the role of TMEM16A for activation of LRRC8/VRAC in HT_29_ cells, which produce large endogenous Ca^2+^-activated Cl^–^ currents and swelling-activated VRAC currents (Centeio et al., 2020; Figure 4). As shown in Figures 1, 2, these experiments were performed with cytosolic-like pipette-filling solution (290 mosm/l, Ca^2+^ 100 nM, pH 7.2; c.f. Methods). The bath was perfused with Ringer solution (300 mosm/l; Ca^2+^ 1.3 mM, pH 7.4). siRNA knockdown of endogenous TMEM16A attenuated hypo-induced whole-cell currents and changed time-independent I_Cl__,swell_ to VRAC-typical time-dependent currents (Figures 4A–D). Notably, the blocker of TMEM16A, niclosamide (1 μM), inhibited I_Cl__,swell_ significantly from 182 ± 22 to 78 ± 9.2 pA/pF (*n* = 5). Knockdown of endogenous LRRC8A strongly attenuated I_Cl__,swell_, while additional knockout of TMEM16A did not further attenuate I_Cl__,swell_. These data suggest that TMEM16A expressed in HT_29_ cells supports activation of LRRC8/VRAC, probably by facilitating hypo-induced Ca^2+^ release from ER Ca^2+^ stores.

**FIGURE 5:**
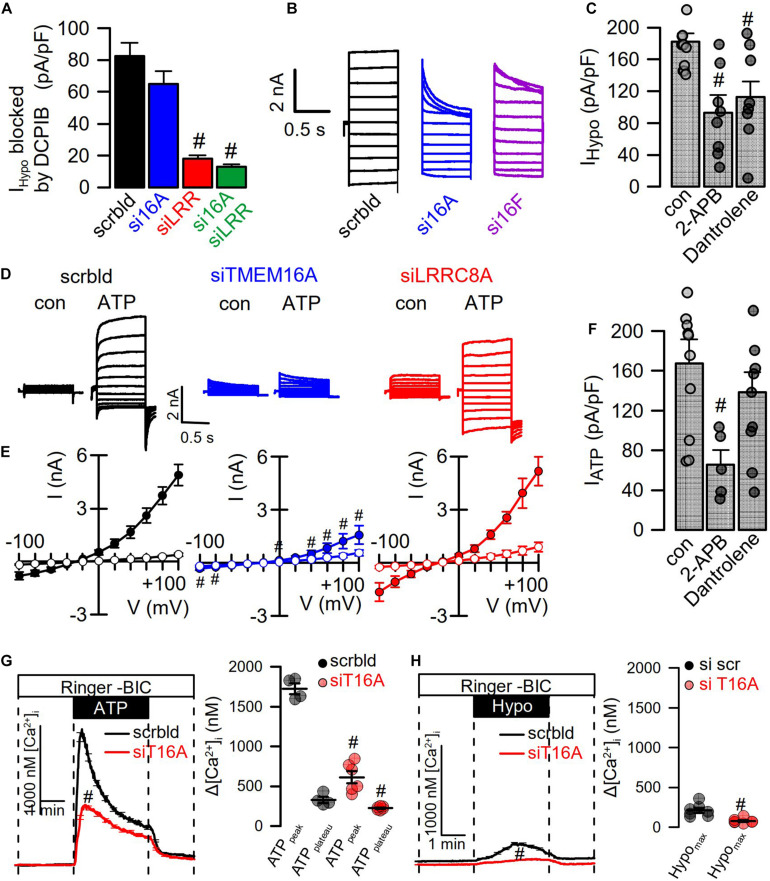
Hypotonicity activates TMEM16A-dependent Ca^2+^ increase. **(A)** Summary of Hypo (200 mosm/l)-induced current densities inhibited by DCPIB in HT_29_ cells (*n* = 9–15). The free Ca^2+^ concentration in the patch pipette filling solution was 100 nM. **(B)** Hypo-induced whole-cell current overlays in HT_29_ cells treated with scrambled RNA (scrbld), siRNA for TMEM16A, or both TMEM16F. **(C)** Effect of 2-APB (50 μM; *p* < 0.001) and dantrolene (50 μM; *p* < 0.001) on Hypo-induced currents densities (Vc = +100 mV) (*n* = 9–11). **(D)** ATP (100 μM)-induced whole-cell current overlays in the absence or presence of siRNAs. **(E)** Corresponding current–voltage relationships (*n* = 9–11). **(F)** Effect of 2-APB (50 μM; *p* < 0.001) and dantrolene (50 μM) on ATP-induced current densities (Vc = +100 mV) (*n* = 7–9). **(G)** Effect of TMEM16A-siRNA on ATP-induced Ca^2+^-increase (*n* = 21–45; *p* < 0.000004 and *p* < 0.01, respectively). **(H)** Effect of TMEM16A-siRNA on Hypo-induced Ca^2+^-increase (*n* = 21–55; *p* < 0.003). Plateau Ca^2+^ was determined at the end of the plateau. Mean ± SEM; ^#^significant decrease (unpaired *t*-test).

The contribution of TMEM16A to swelling activation of endogenous VRAC was examined in mock-transfected HEK293 cells (-T16A) or HEK293 cells overexpressing TMEM16A (+T16A) at different extracellular hypotonicities. VRAC was activated by extracellular bath solution of different hypotonicities (275, 240, 200, and 150 mosm/l). The data demonstrate an increase in hypotonic activation of VRAC by coexpression of TMEM16A, which was more significant at less severe hypotonicity (Supplementary Figure S1). These experiments were performed at 10 nM intracellular Ca^2+^ concentration.

### Contribution of TMEM16A to I_Cl__,swell_ Under Non-voltage Clamp Conditions

**FIGURE 6:**
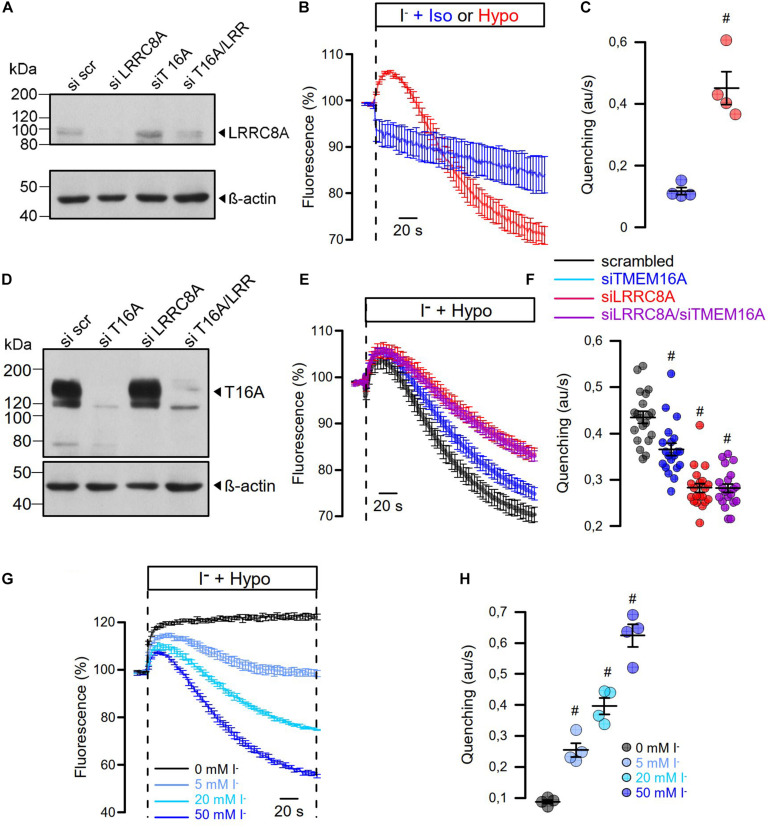
Hypo-induced anion permeability measured under non-voltage clamp conditions. **(A)** siRNA knockdown of LRRC8A detected by Western blotting. The knockdown efficiency was 72%. Knockdown of TMEM16A enhanced the expression of LRRC8A, corresponding to Benedetto et al. (2016). **(B)** Summary of time dependence for YFP quenching induced by 20 mM iodide in the presence of isotonic Ringer (Iso; 300 mosmol/l) or Hypo (200 mosm/l). **(C)** Summary of initial rate of quenching (arbitrary units/s) (*n* = 4; *p* < 0.006). **(D)** siRNA knockdown of TMEM16A detected by Western blotting. **(E)** Summary of time dependence for Hypo-induced YFP quenching in the absence or presence of siRNA. **(F)** Summary of initial rates of quenching (arbitrary units/s) (*n* = 19). *p* < 0.0005 (siTMEM16A), *p* < 2 × 10^–11^ (siLRRC8A), *p* < 2 × 10^–11^ (siLRRC8A/siTMEM16A). **(G,H)** Hypo-induced YFP quenching was absent in the presence of 0 mM extracellular iodide but was gradually increased with 5 mM (*n* = 4; *p* < 0.003), 20 mM (*n* = 4; *p* < 0.0009), and 50 mM (*n* = 4; *p* < 0.0005). Mean ± SEM; ^#^significant increase or decrease, respectively (unpaired *t*-test). Blots were done as replicates.

As cell swelling and volume regulation take place under non-voltage clamp conditions, we examined the role of TMEM16A for activation of I_Cl__,swell_ in iodide quenching experiments using halide-sensitive yellow fluorescent protein (YFP). When applying hypotonic bath solution (200 mosm/l), immediate increase in YFP-fluorescence is expected due to rapid uptake of water through aquaporins, dilutions of anions, and de-quenching of YFP fluorescence, which occurs within seconds (Benedetto et al., 2016). This is followed by iodide-induced quenching, as VRAC channels are swelling activated, thus allowing entry of iodide (Figure 6B). Analysis of the maximal rate of quenching showed a clear activation of halide conductance by Hypo (Figure 6C). siRNA knockdown of TMEM16A or LRRC8A (Figures 6A,D) showed similar results as in patch-clamp experiments: Knockdown of TMEM16A attenuated the Hypo-induced quenching, while it did not further reduce quenching inhibited by siLRRC8A (Figures 6E,F). It should be noted that in previous studies we observed an inverse correlation between expression of TMEM16A and LRRC8A. For example, TMEM16A was found to be strongly upregulated in cells that lacked expression of LRRC8A (Benedetto et al., 2016). Thus, it may not be surprising to find that LRRC8A was less efficient downregulated with parallel knockdown of TMEM16A (Western blot in Figure 6A).

### Hypotonicity Activates TMEM16A-Dependent Ca^2+^ Increase

When analyzing instantaneous peak current densities, the VRAC blocker DCPIB inhibited I_Cl__,swell_ independent of siRNA-TMEM16A, while siRNA-LRRC8A strongly inhibited instantaneous peak currents (Figure 5A). However, knockdown of TMEM16A induced pronounced time-dependent inactivation of I_Cl__,swell_, and the same was observed for knockdown of TMEM16F (Sirianant et al., 2016a; Figure 5B). Notably, TMEM16F has also been reported to conduct Ca^2+^ ions, apart from its ability to scramble phospholipids (Yang et al., 2012). Thus, TMEM16 proteins maintain I_Cl__,swell_ activity, possibly by supporting ER Ca^2+^ release and through activation of Ca^2+^ influx (Benedetto et al., 2016; Cabrita et al., 2017). To further elucidate the contribution of Ca^2+^ store release to activation of I_Cl__,swell_, cells were swollen in the presence of IP3R-inhibitor 2-ABP or the RyR inhibitor dantrolene, which both inhibited I_Cl__,swell_ (Figure 5C). Activation of CaCC in HT_29_ cells by the purinergic ligand ATP was entirely dependent on TMEM16A, as shown by siRNA-TMEM16A, and knockdown of LRRC8A did not compromise activation of CaCC (Figures 5D,E). This indicates that Ca^2+^ increase alone is not sufficient to activate VRAC/LRRC8A. Unlike stimulation of I_Cl__,swell_, activation of CaCC was only inhibited by 2-ABP, but not by dantrolene (Figure 5F). Using the Ca^2+^ sensor Fura-2, we examined how TMEM16A affects Ca^2+^ increase induced by ATP and Hypo (200 mosm/l). ATP induced a typical peak (ER-Ca^2+^ store release) and plateau (Ca^2+^ influx; SOCE) response, which were both inhibited in the absence of TMEM16A (Cabrita et al., 2017; Figure 5G). The Hypo-induced Ca^2+^ increase was much smaller but was also inhibited by siRNA-TMEM16A (Figure 5H). Taken together, the data demonstrate the role of TMEM16A for swelling-induced Ca^2+^ increase, which is important for full activation of I_Cl__,swell_.

In additional experiments, we varied the iodide concentration in the hypotonic bath solution between 0 and 50 mM (Figures 6G,H). It is shown that at 0 mM iodide, no quenching takes place but only cell swelling, as indicated by an increase in fluorescence. With increasing iodide concentrations in the extracellular buffer, the maximum of YFP dequenching is no longer reached and the rate of YFP quenching increases. The data suggest a very fast activation of VRAC, which probably parallels the decrease in intracellular ionic strength (Syeda et al., 2016). In contrast, the regulatory volume decrease (RVD) takes considerably longer as indicated by the delayed YFP—re-quenching in the presence of 0 mM iodide.

**FIGURE 7:**
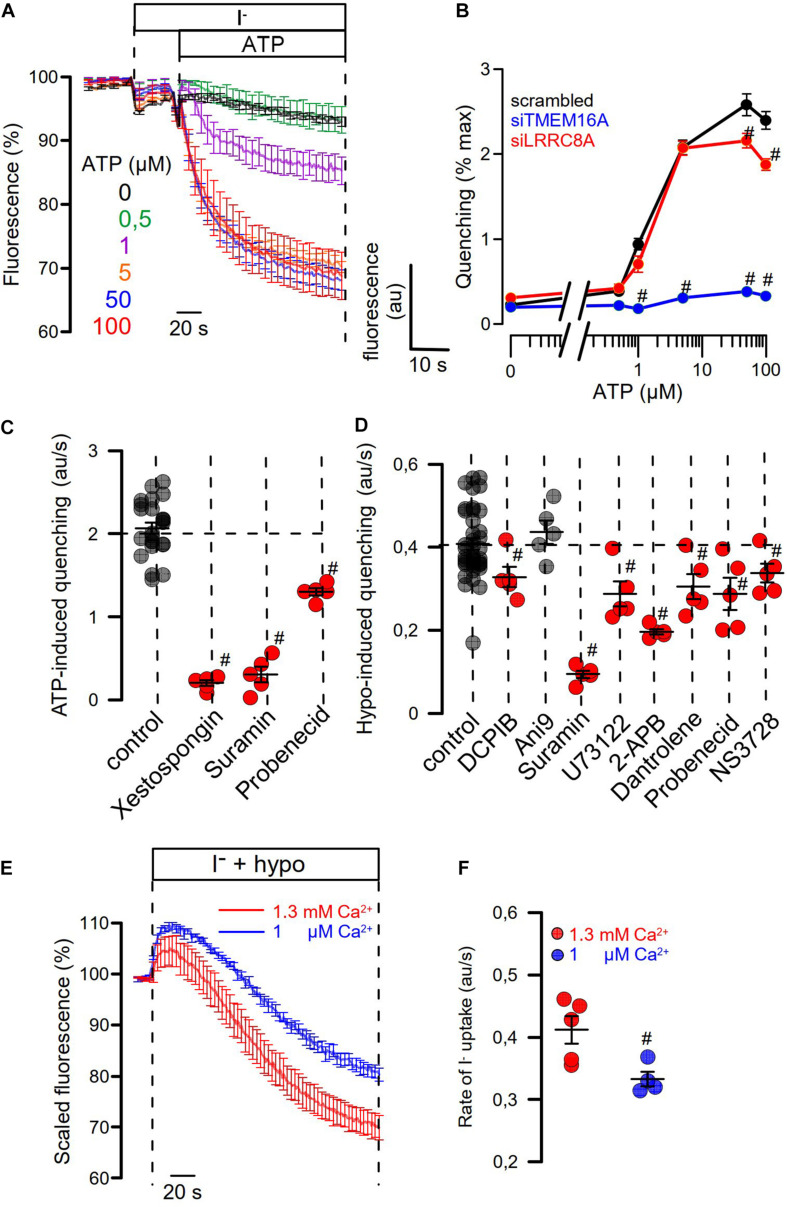
Role of Ca^2+^ signaling for Hypo- and ATP-induced anion permeability. **(A)** Time-dependent YFP quenching induced by different concentrations of ATP (*n* = 3–5). **(B)** Concentration dependence of ATP-induced quenching (arbitrary units/s) and effects of knockdown of TMEM16A or LRRC8A (*n* = 4). **(C)** Summary of ATP-induced quenching in the absence (control) or presence of IP3R blockers xestospongin C (10 μM; *p* < 10^–19^), suramin (500 μM; *p* < 10^–7^), and probenecid (1 mM; *p* < 2 × 10^–9^) (*n* = 4–5). **(D)** Summary of Hypo (200 mosm/l)-induced quenching in the absence (control) or presence of DCPIB (30 μM; *p* < 0.02), Ani9 (10 μM), suramin (500 μM; *p* < 2 × 10^–7^), U73122 (10 μM; *p* < 0.01), 2-APB (50 μM; *p* < 10^–17^), dantrolene (50 μM; *p* < 0.02), probenecid (1 mM; *p* < 0.03), and NS3728 (10 μM; *p* < 0.03) (*n* = 4–5). **(E,F)** The contribution of Ca^2+^ influx on Hypo-induced quenching was examined by exposing the cells to a hypotonic solution in the presence of physiological (1.3 mM; *n* = 5) or low (1 μM; *n* = 4; *p* < 0.02) extracellular Ca^2+^ concentration. Mean ± SEM; ^#^significant inhibition (unpaired *t*-test).

We examined concentration-dependent quenching by ATP and found pronounced inhibition of quenching by siRNA-TMEM16A (Figures 7A,B). Notably, at pronounced stimulation with saturating concentrations of ATP (50, 100 μM), ATP-induced quenching was slightly but significantly inhibited by siLRRC8A. This suggests a contribution of VRAC to ATP-induced halide permeability. We examined the effects of a number of different inhibitors on activation of I_Cl__,swell_ by testing them individually in HT_2__9_ cells. We decided to analyze the effects of the various inhibitors in YFP-quenching experiments with non-dialyzed cells and under non-voltage clamp conditions (instead of whole-cell patch clamping), to leave intracellular Ca^2+^ signaling untouched and to avoid artifacts due to voltage clamping.

Three different inhibitors of IP3R-mediated ER Ca^2+^-store release, xestospongin C, suramin, and probenecid, blocked ATP-induced activation of halide permeability (Figure 7C). Hypo (200 mosm/l)-induced quenching was blocked by the VRAC blocker DCPIB, but not by the TMEM16A blocker Ani9 (Seo et al., 2016), suggesting that the presence of TMEM16A, but not its Cl^–^ conductance, supports activation of I_Cl__,swell_ (Figure 7D). Surprisingly, the inhibitory effect of DCPIB on Hypo-induced quenching, i.e., activation of VRAC, was rather weak. However, this is probably explained by the strong voltage dependence of VRAC inhibition by DCPIB. During YFP measurements, DCPIB was applied under non-voltage clamp conditions, i.e., at the intrinsic negative membrane voltage of the HT_29_ cells, which is around −40 mV. In additional patch-clamp experiments, we found indeed weak inhibition of VRAC by DCPIB at negative clamp voltages but a much more pronounced inhibition at depolarized clamp voltages (Supplementary Figure S2). The cystic fibrosis transmembrane conductance regulator (CFTR), another Cl^–^ channel expressed in HT_29_ cells, is unlikely to contribute to I*_Cl__,swell_*, as the CFTR inhibitor CFTRinh172 (30 μM) did not inhibit activation of VRAC (data not shown).

A number of compounds inhibiting IP3R- and RyR-mediated ER Ca^2+^-store release, such as suramin, U73122, probenecid, and dantrolene, as well as NS3728 (Helix et al., 2003), inhibited activation of I_Cl__,swell_. HT_2__9_ cells were found to express all three paralogs of the IP3 receptor as well as the ryanodine receptor RyR2, along with the TRPV4 channel, which is the Ca^2+^ influx channel most frequently found to have a role in volume regulation (Pasantes-Morales, 2016; Supplementary Figure S3). The contribution of Ca^2+^ influx for activation of VRAC was demonstrated by exposing the cells to hypotonic solution in the presence of low (1 μM) extracellular Ca^2+^, which attenuated the activation of VRAC (Figures 7E,F). Taken together, Ca^2+^ may not be a prerequisite for activation of VRAC. However, Ca^2+^ store release and Ca^2+^ influx facilitate its activation, which is in line with earlier observations (Akita et al., 2011; Sirianant et al., 2016a; Liu et al., 2019). TMEM16A facilitates intracellular compartmentalized Ca^2+^ increase and thus supports activation of VRAC. Along with swelling activation of VRAC, TMEM16A is activated through swelling-induced rise in intracellular Ca^2+^. The extent of this coregulation of I*_Cl__,swell_* is cell dependent and depends on expression of TMEM16 proteins.

## Discussion

The present study examines activation of I*_Cl__,swell_* in two different cell lines. HEK293 cells were used because these cells do not express TMEM16A and express only very low levels of LRRC8A. Thus, the cell line was ideal to examine the effects of overexpressed TMEM16A and LRRC8A/C. In contrast, HT_29_ cells express both TMEM16A and LRRC8A and show large ATP-stimulated, i.e., Ca^2+^ activated TMEM16A Cl^–^ currents and pronounced swelling-activated LRRC8/VRAC Cl^–^ currents. LRRC8A (VRAC) and TMEM16A (CaCC) are independent molecular entities and Cl^–^ channels activated by cell swelling or intracellular Ca^2+^. Although TMEM16 proteins are not VRACs *per se*, previous data suggest a role in I_Cl__,swell_ (Almaca et al., 2009; Juul et al., 2014; Benedetto et al., 2016; Sirianant et al., 2016a; Liu et al., 2019; Zhang et al., 2020). Analysis of I_Cl__,swell_ in tissues from mice lacking expression of TMEM16A (Almaca et al., 2009), TMEM16F (Ousingsawat et al., 2015; Sirianant et al., 2016a), and TMEM16K (Hammer et al., 2015; Wanitchakool et al., 2017) shows reduced I_Cl__,swell_) and regulatory volume decrease (RVD). The contribution of TMEM16A and other TMEM16-proteins to I_Cl__,swell_ and volume regulation is cell dependent and may be particularly relevant in highly differentiated native (non-cultured) cells. Equally important appear the patch-clamp conditions under which I_Cl__,swell_ are measured, which may explain some of the earlier controversial findings regarding the role of TMEM16F (Almaca et al., 2009; Shimizu et al., 2013; Juul et al., 2014; Sirianant et al., 2016a).

Patch-clamp experiments showed more impressive effects of TMEM16A (and TMEM16F) on activation of VRAC at depolarized clamp voltages. TMEM16A and TMEM16F mainly abolish time-dependent inactivation of VRAC. This has also been described in detail in a previous study (Sirianant et al., 2016a). Thus, the impact of TMEM16A is less remarkable at negative clamp voltages and under non-voltage clamp conditions. Nevertheless, even at negative membrane voltages the impact of TMEM16A was found to be significant (Figure 6 and Supplementary Figure S4). While a number of transport properties were ascribed to LRRC8/VRAC (Planells-Cases et al., 2015; Lutter et al., 2017; Kang et al., 2018; Stuhlmann et al., 2018; Zhou et al., 2020), its physiological relevance in terms of volume regulation is still a matter of debate. We found that cells are able to perform RVD also in the absence of LRRC8A (Milenkovic et al., 2015; Benedetto et al., 2016; Sirianant et al., 2016b), while other different members of the TMEM16A family, CFTR, and the KCl cotransporter KCC clearly contribute to volume regulation (Sirianant et al., 2016b; Wanitchakool et al., 2016). We found that activation of I_Cl__,swell_ was fast (within 1 min), which, however, was still somewhat delayed when compared to immediate cell swelling (within seconds) (Benedetto et al., 2016). This suggested additional regulatory steps, apart from direct opening of VRAC by low ionic strength (Syeda et al., 2016). Others and we proposed Ca^2+^-dependent unfolding of caveolae-like membrane reserves upon cell swelling and activation of I_Cl__,swell_ (Groulx et al., 2006; Kozera et al., 2009; Benedetto et al., 2016).

This additional mechanism may require Ca^2+^ increase in an intracellular compartment close to the plasma membrane (Akita et al., 2011; Benedetto et al., 2016; Liu et al., 2019). Liu et al. showed that intracellular Ca^2+^ was necessary but not sufficient to activate LRRC8A-mediated currents (Liu et al., 2019). Lemonnier and coworkers provided evidence for a colocalization of VRAC with store-operated Ca^2+^ channels and showed that activation of VRAC was strongly dependent on Ca^2+^ release through IP3R (Lemonnier et al., 2002). They concluded that VRAC is regulated within Ca^2+^ microdomains. Similarly, Akita and collaborators suggested that VRAC/VSOR channels can be activated by PLC-coupled GPCRs, which depends on Ca^2+^ store release in close vicinity of the channel (Akita and Okada, 2011) and proposed a Ca^2+^ nanodomain-mediated component of VRAC (Akita et al., 2011).

Our present data may help to clarify the role of TMEM16 proteins for Ca^2+^-dependent activation of VRAC. The role of TMEM16A and other members of the TMEM16-family for ER Ca^2+^ release was found meanwhile in numerous tissues (Jin et al., 2016; Cabrita et al., 2017; Wanitchakool et al., 2017; Benedetto et al., 2019a; Centeio et al., 2020; Park et al., 2020). TMEM16A-controlled Ca^2+^ release is also essential for activation of CFTR (Benedetto et al., 2017, 2019b; Park et al., 2020). Thus, cell swelling-induced Ca^2+^ release from the ER and activation of VRAC is facilitated in the presence of TMEM16 proteins. Because knockdown of TMEM16A but not inhibition of TMEM16A by Ani9 attenuated activation of VRAC, it suggests that ER-tethering by TMEM16A rather than Cl^–^ transport supports activation of VRAC (Jin et al., 2013; Cabrita et al., 2017).

Along this line, TMC8, a member of the closely related family of transmembrane channel-like TMC proteins, also controlled activation of VRAC and volume regulation (Sirianant et al., 2014). Depending on the cell type, swelling-induced Ca^2+^ release will activate TMEM16 proteins and CFTR (Thiele et al., 1998; Wanitchakool et al., 2016), which parallels activation of VRAC. This circumstance may explain many earlier findings, such as the overlapping Cl^–^ channel pharmacology (Koslowsky et al., 1994; Rottgen et al., 2018; Centeio et al., 2020).

## Data Availability Statement

The raw data supporting the conclusions of this article will be made available by the authors, without undue reservation, to any qualified researcher.

## Author Contributions

RC, JO, and RS performed the experiments and analyzed the data. RC, RS, and KK wrote the manuscript. All authors contributed to the article and approved the submitted version.

## Conflict of Interest

The authors declare that the research was conducted in the absence of any commercial or financial relationships that could be construed as a potential conflict of interest.
