# Supplementary material for: Ca2+ Dependence of Volume-Regulated VRAC/LRRC8 and TMEM16A Cl– Channels
Source: Front Cell Dev Biol. 2020 Dec 1;8:596879. doi: 10.3389/fcell.2020.596879 (PMC7736618; doi:10.3389/fcell.2020.596879)
Supplement: Supplementary file 1 [file Data_Sheet_1.PDF]

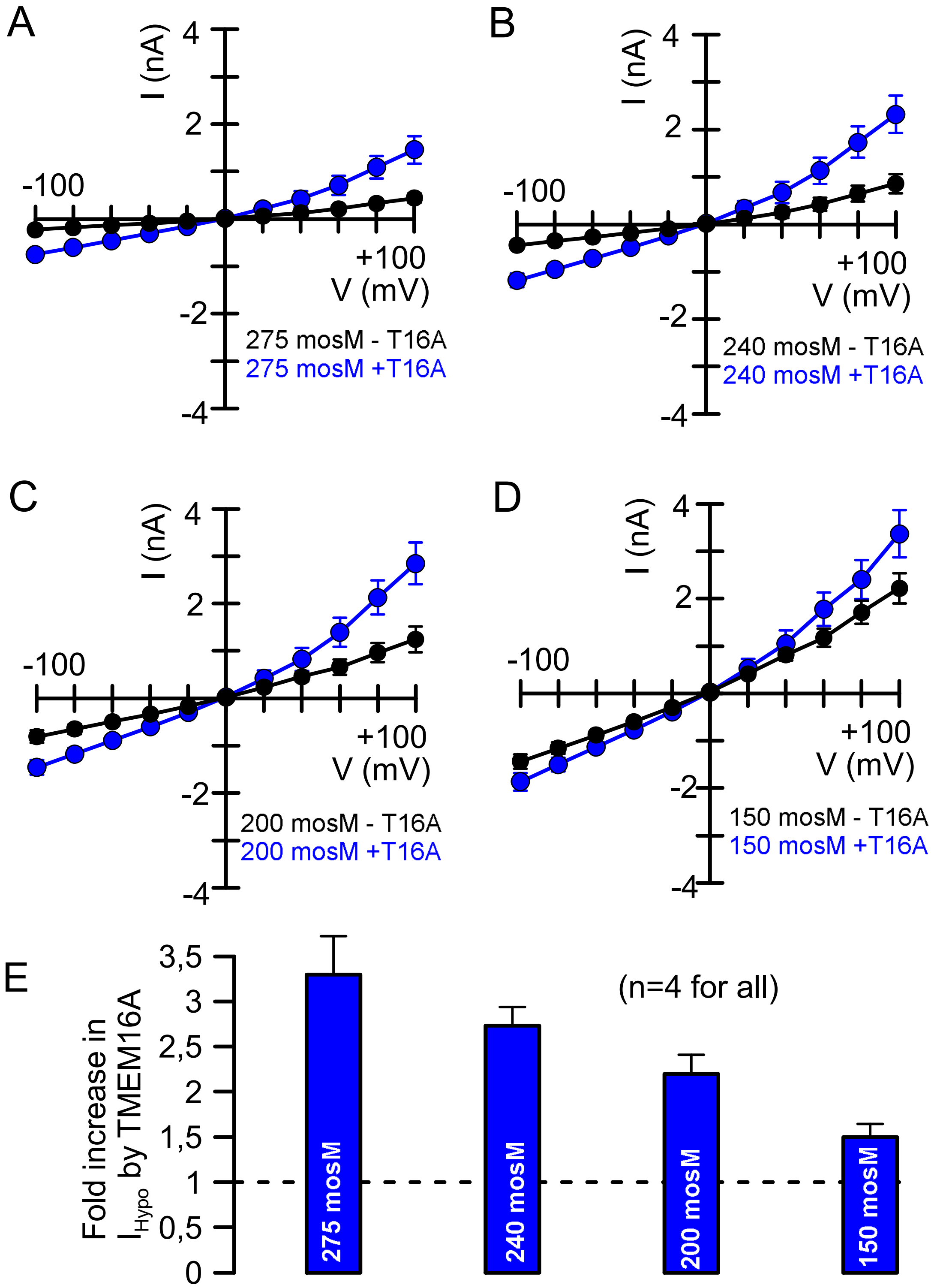

Supplementary Fig. 1: Contribution of TMEM16A to activation of VRAC at different extracellular hypotonicity. The contribution of TMEM16A to swelling activation of endogenous VRAC was examined in mock-transfected HEK293 cells (-T16A), or HEK293 cells overexpressing TMEM16A (+T16A). VRAC was activated by extracellular bath solution of different hypotonicity (275 mosm/l, 240 mosm/l, 200 mosm/l, 150 mosm/l). Osmolarities were adjusted by removing adequate amounts of NaCl from Ringer solution. A-D) Current/voltage relationships for VRAC activated by 275 mosm/l, 240 mosm/l, 200 mosm/l, and 150 mosm/l in the absence (black curve) or presence (blue curve) of TMEM16A. E) Fold increase in hypotonic activation of VRAC by coexpression of TMEM16A at different extracellular hypotonicity demonstrates a more significant effect of TMEM16A on the activation of VRAC at less severe hypotonicity. Experiments were performed at 10 nM intracellular Ca^{2+} concentration. Because there is no activation of TMEM16A at 10 nM intracellular Ca^{2+} concentration, there is probably no synergistic or additive effect of VRAC and TMEM16A. The data suggest that the presence of TMEM16A potentiates activation of VRAC by hypotonic cell swelling.
