# Supplementary material for: Ca2+ Dependence of Volume-Regulated VRAC/LRRC8 and TMEM16A Cl– Channels
Source: Front Cell Dev Biol. 2020 Dec 1;8:596879. doi: 10.3389/fcell.2020.596879 (PMC7736618; doi:10.3389/fcell.2020.596879)
Supplement: Supplementary file 2 [file Data_Sheet_2.pdf]

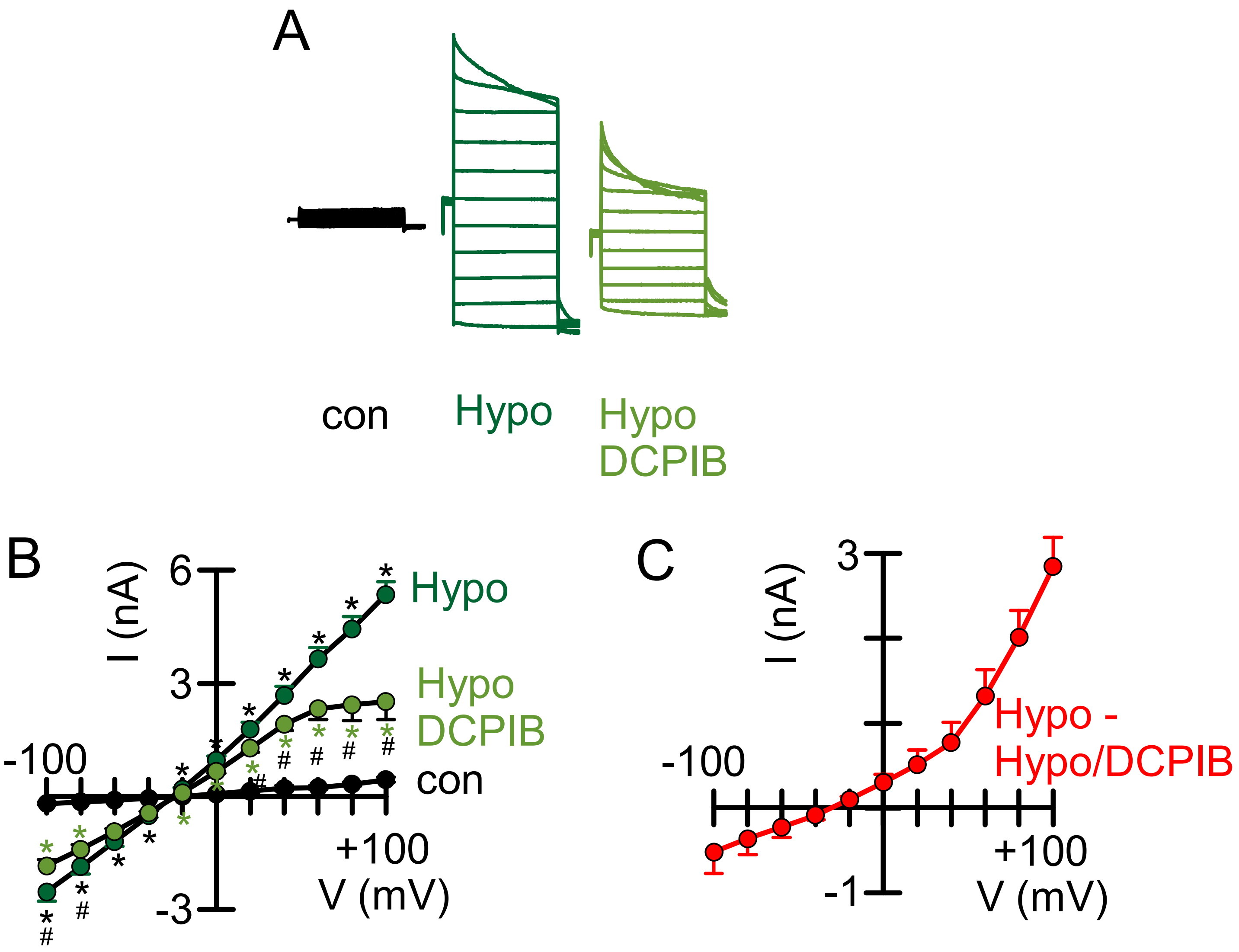

Supplementary Fig. 2: A) Whole cell patch clamp recordings showing activation of VRAC in HT_{29} cells by hypotonic (200 mosm/l) cell swelling. VRAC was inhibited by DCPIB at depolarized clamp voltages but showed relatively little effect at negative clamp voltages. B) Corresponding current voltage relationships. Mean ± SEM (n = 6). C) I/V curve for remaining currents after subtracting $I_{Hypo/DCPIB}$ from I_{Hypo}. *significant activation by Hypo (paired t-test, $p<0.0000007$ for both). #significant inhibition by DCPIB (paired t-test, $p<0.0006$).
