# Supplementary material for: Ca2+ Dependence of Volume-Regulated VRAC/LRRC8 and TMEM16A Cl– Channels
Source: Front Cell Dev Biol. 2020 Dec 1;8:596879. doi: 10.3389/fcell.2020.596879 (PMC7736618; doi:10.3389/fcell.2020.596879)
Supplement: Supplementary file 3 [file Data_Sheet_3.pdf]

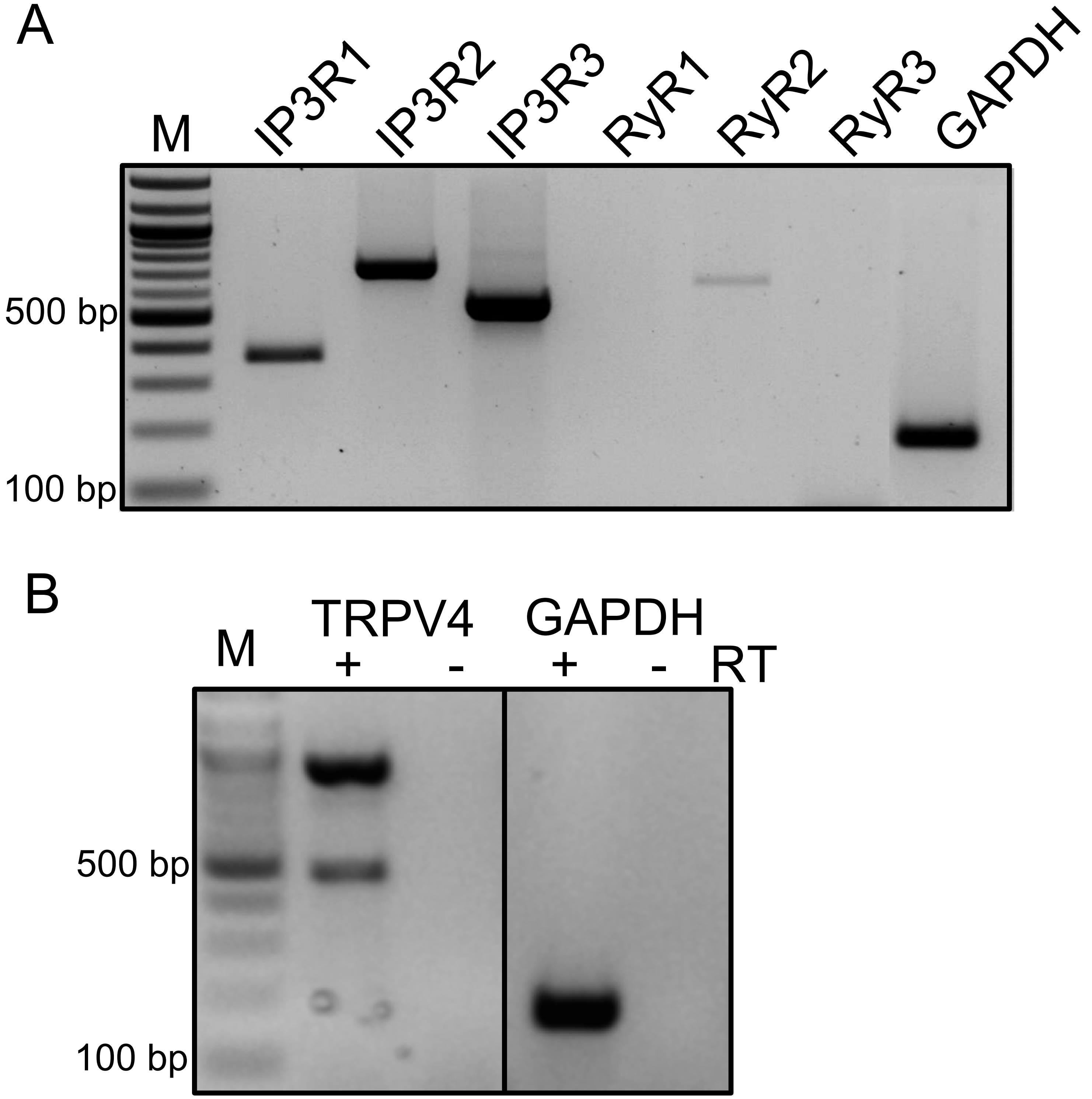

Supplementary Fig. 3: RT-PCR analysis of the expression of different paralogs of IP_3 receptors and ryanodine receptors (A) and TRPV4 (B) in HT_{29} cells.
