# Supplementary material for: Ca2+ Dependence of Volume-Regulated VRAC/LRRC8 and TMEM16A Cl– Channels
Source: Front Cell Dev Biol. 2020 Dec 1;8:596879. doi: 10.3389/fcell.2020.596879 (PMC7736618; doi:10.3389/fcell.2020.596879)
Supplement: Supplementary file 4 [file Data_Sheet_4.PDF]

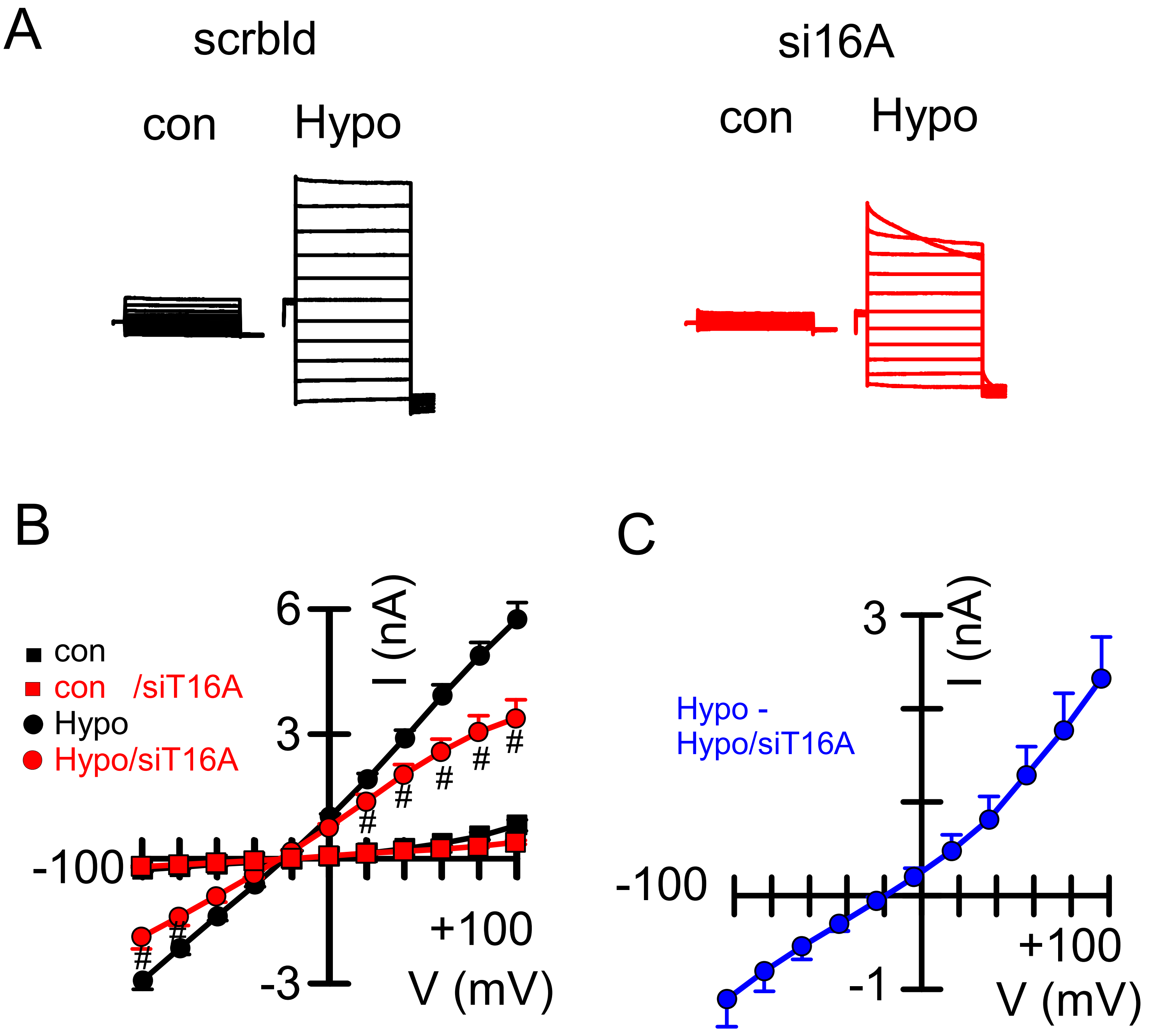

Supplementary Fig. 4: A) Whole cell patch clamp recordings showing activation of VRAC in HT_{29} cells, by hypotonic (Hypo; 200 mosm/l) cell swelling in the presence or absence of TMEM16A (siT16A). B) Corresponding current voltage relationships indicating reduced VRAC-currents in the positive and negative voltage range in the absence of TMEM16A (siTMEM16A). C) I/V curve for remaining currents after subtracting $I_{Hypo/siT16A}$ from I_{Hypo}. Mean ± SEM (n = 7 for all). #significant inhibition by siT16A (unpaired t-test, p<0.03).
